# Exploring the Solubility and Bioavailability of Sodium Salt and Its Free Acid Solid Dispersions of Dolutegravir

**DOI:** 10.1155/2023/7198674

**Published:** 2023-06-20

**Authors:** Dani Lakshman Yarlagadda, Akshatha M. Nayak, Bheemisetty Brahmam, Krishnamurthy Bhat

**Affiliations:** ^1^Department of Pharmaceutical Quality Assurance, Manipal College of Pharmaceutical Sciences, Manipal Academy of Higher Education (MAHE), Manipal, Karnataka 576104, India; ^2^Department of Pharmaceutics, Manipal College of Pharmaceutical Sciences, Manipal Academy of Higher Education (MAHE), Manipal, Karnataka 576104, India

## Abstract

Amorphous salt solid dispersion (ASSD) of Dolutegravir amorphous salt (DSSD) was generated using quench cooling and compared to its Dolutegravir free acid solid dispersion (DFSD) to improve the solubility and bioavailability. Soluplus (SLP) was used as a polymeric carrier in both solid dispersions. The prepared DSSD and DFSD, physical mixtures, and individual compounds were characterized by employing DSC, XRPD, and FTIR to assess the formation of the single homogenous amorphous phase and the existence of intermolecular interactions. Partial crystallinity was observed for DSSD, unlike DFSD, which is completely amorphous. No intermolecular interactions were observed between the Dolutegravir sodium (DS)/Dolutegravir free acid (DF) and SLP from the FTIR spectra of DSSD and DFSD. Both DSSD and DFSD improved the solubility of Dolutegravir (DTG) to 5.7 and 4.54 folds compared to the pure forms. Similarly, drug release from DSSD and DFSD was 2 and 1.5 folds higher than that in the pure form, owing to the rapid dissolution of the drug from the formulations. The permeability of DSSD and DFSD was estimated using the dialysis membrane, which enhanced the DTG permeability. The improvement in *in vitro* studies was translated into *in vivo* pharmacokinetic profiles of DSSD and DFSD, where 4.0 and 5.6 folds, respectively, improved the *C*_max_ of DTG.

## 1. Introduction

The relevance of enviable physicochemical properties in formulation development, including solubility and permeability, cannot be overstated [1]. The poor water solubility for molecules affects about 40% and 90% of marketed and developmental drugs in the pharmaceutical formulation [2, 3]. This frequently results in challenges with oral absorption, followed by inadequate blood concentrations to produce the desired therapeutic effects [4]. In light of this, various formulation techniques have been introduced to augment the solubility of BCS class II and IV drugs, such as particle size reduction [5], salt formation [6], cocrystal [7], and lipid-based formulations [8]. Albeit of solubility improvement, each of these techniques has respective advantages and limitations, often reliant on the physicochemical composition of the molecules being studied [9]. Particle size reduction is not desirable when drug wettability is poor [10]. Further, the drug may undergo thermal and physical stress during the particle size reduction of drug substances via mechanical methods like milling, which may result in degradation and recrystallization [11]. Lipid solution-based approaches are usually limited to potent and highly lipophilic molecules [12]. Though the salt form of drugs increases the drug release rate and solubility, salt formation of all compounds is not sustainable. Moreover, the main disadvantage is that salt gets precipitated and converted to its respective free acid/base form when it is orally administered [13].

Amorphization of drugs has been developed as a viable bioavailability improved technique for poorly water-soluble drugs due to the supersaturation-driven solubility advantage of the amorphous form [14]. The transformation from crystalline to an amorphous state resulted in the improved thermodynamic activity of the drug, thereby improving drug solubility. Nevertheless, physical stability remains an issue for an amorphous form of drugs often caused due to higher molecular mobility [15]. Polymeric solid dispersions (SDs), popularly known as amorphous solid dispersions (ASDs), have been established as one of the most efficient methods for preventing amorphous drug crystallization and phase separation both in the solid as well as liquid phases of the drug due to molecular level dispersion of drug in the polymers [16]. The ASDs improve drug solubility through different mechanisms, either in combination or alone, including amorphization, wettability, particle size reduction, inhibition of drug recrystallization, and intermolecular interactions [17, 18]. Furthermore, in the preparation of ASDs, techniques such as hot-melt extrusion [19], spray drying [20], quench cooling [21], solvent evaporation [22], and freeze-drying [23] were widely utilized. Amorphous salt solid dispersion (ASSD) is a new approach that combines the advantages of amorphization and salt formation to improve the physical stability, aqueous solubility, and dissolution rate of the drug in an amorphous form. Since electrostatic interactions are more substantial than hydrogen bonding interactions, the formation of salt with either counterions or excipients stabilizes the drug in amorphous form [24, 25]. Mesallati and Tajber prepared Ciprofloxacin ASSD using succinic acid along with polymers and observed the improvement in solubility and physical stability for ASSD compared to pure forms and ASDs [26].

Dolutegravir (DTG) is a human immunodeficiency virus type 1 (HIV-1) integrase inhibitor, which is combined with other antiretroviral agents for treating HIV-1 infection [27]. It is classified as BCS class II and has a low aqueous solubility [28, 29]. The pKa of DTG is 8.2 and 10.1, which is weakly acidic; therefore, DTG remains primarily in a unionized form and, thus, poorly soluble all over the gastrointestinal tract (GIT). Because of these physicochemical properties, DTG is used as an ideal compound to prepare ASSD. Interestingly, no methodological studies are available attempting DTG amorphous salt to prepare for ASSD. In this regard, the primary objective of the current study was to concurrently enhance the solubility and bioavailability of Dolutegravir amorphous salt (DS) by leveraging the ASSD strategy. DTG is an appropriate molecule for this strategy due to its poor aqueous solubility and ability of salt formation [30, 31]. The ASSD of DTG was prepared using the quench cooling technique with Soluplus® (SLP) as a polymeric carrier. The secondary aim of the present study was to evaluate the solubility, *in vitro* drug release, permeability, and *in vivo* pharmacokinetics of DSSD to those of free acid solid dispersion DFSD.

## 2. Materials and Methods

### 2.1. Materials

Amorphous Dolutegravir sodium (DS) was obtained from STEERLife Pvt. Ltd., Bengaluru, India, and Hetero Drugs Limited as a gift sample (Supplementary Figure 1(a)). The polyvinyl caprolactam-polyvinyl acetate-polyethylene glycol graft copolymer, i.e., Soluplus® (SLP), was procured from BASF, Germany, as a gift sample. Solvents like methanol and acetonitrile of HPLC grade were acquired from Merck Life Sciences Pvt. Ltd., Mumbai, India. Ultra-pure water was acquired from the Siemens Water Purification System in the lab.

### 2.2. Methods

#### 2.2.1. Preparation of DTG Solid Dispersions

Solid dispersions of amorphous Dolutegravir sodium (DSSD) and Dolutegravir free acid (DFSD) were prepared at 20 : 80% w/w drug-polymer ratio. DS/Dolutegravir free acid (DF) and SLP of the above ratio were weighed in a silica crucible and mixed physically. The mixture was then heated up to 240°C. Further, the crucible with molten mass was instantaneously transferred to a glass desiccator and stored for 3 hours at −80°C. After 3 hours, the crucible was taken out, and the melt was further powdered and stored in a vacuum desiccator for further use.

#### 2.2.2. Preparation of Dolutegravir Free Acid

DS of 500 mg was dissolved in 50 mL of water at 20–25°C. The pH of DS solution was adjusted to 1.50 and maintained for 60 minutes at 20–25°C to obtain the free acid form of Dolutegravir (DF) (Supplementary Figure 1(b)). The precipitate was filtered and stored for 10–12 hours in a vacuum desiccator at 45°C for future use [32]. The prepared DF was stable at this temperature [33].

#### 2.2.3. Analytical Technique

A Prominence HPLC system containing an LC-20AD pump, SIL-20AC auto-injector, and SPD-20A UV-VIS detector was used for the determination of DTG. Chromatographic separation of DTG was performed on Phenomenex C18 column (250 × 4.6 mm, 5 *μ*m) by isocratic elution mode using the acetonitrile and 25 mM phosphate buffer (pH 3.5) as mobile phase at 45:55 %v/v ratio, and the flow rate was adjusted to 1 mL/min with 20 *μ*L injection volume and the eluents were detected at 258 nm. The protein precipitation extraction technique was employed to estimate DTG in plasma samples using an identical amount of chilled acetonitrile. Raloxifene HCl was treated as an internal standard. Furthermore, the bioanalytical method was validated in accordance with the USFDA protocol, and parameters are presented in the supplementary material section along with the representative chromatograms (Figures 2 and 3) [34]. The validated method showed a retention time of 11.1 minutes and had linearity from 350 to 7000 ng/mL (*R*^2^ = 0.9980). The lower limit of detection of the method was found to be 350 ng/mL.

#### 2.2.4. Assay of DSSD and DFSD

The drug content of DTG in the produced amorphous Dolutegravir sodium solid dispersion (DSSD) and Dolutegravir free acid solid dispersion (DFSD) was evaluated using the aforementioned analytical procedure. Standard and test samples were prepared in triplicate by dissolving 10 mg DSSD and DFSD powder corresponding to 10 mg DTG in 10 mL methanol. Serial dilutions with 50 : 50 methanol and water were used to obtain a 1 *μ*g/mL sample. The results were compared to a 1 *μ*g/mL DTG standard.

#### 2.2.5. Solid State Characterization

*(1) Differential Scanning Calorimetry (DSC)*. Shimadzu DT-60 apparatus was employed for performing the thermal analysis. In an aluminium pan crimped with a lid, 5 mg of pure DS, DF, and produced DSSD and DFSD were loaded. Powdered alumina was used as a reference which is also crimped as above. The sample was set in a sample holder and operated at 10°C/min from 25°C to 300°C under nitrogen flow.

*(2) Fourier-Transform Infrared Spectroscopy (FTIR)*. The prepared DSSD and DFSD FTIR spectra were obtained using a Shimadzu FTIR IRAffinity-1 spectrophotometer over a range from 4000 to 500 cm^−1^ (25 scans, 4 cm^−1^). Each sample was dispersed in KBr and ground with a motor and pestle before being formed into a disc under the pressure of about 1000 psig.

*(3) X-Ray Powder Diffraction (XRPD)*. The X-ray powder diffractograms were recorded by using a Rigaku MiniFlex 600 X-ray diffractometer. The instrument was operated at 15 mA and 40 kV of current and voltage. Scanning at 5–40° (2*θ*) was used to measure the diffraction intensities.

#### 2.2.6. Solubility Determination

The shake flask method determined the solubility of DS, DF, physical mixture (PM), and DSSD and DFSD. An excess amount of prepared dispersions and pure DS and DF were added to solubility vials containing pH 6.8 phosphate buffer. The samples were placed in the orbital shaker at 37°C for 24 hours at 150 rpm to achieve equilibrium. A Remi C24 centrifuge was used to centrifuge the samples for 10 minutes at 10000 rpm and 4°C. The collected supernatant was diluted and analyzed using HPLC.

#### 2.2.7. *In Vitro* Dissolution Studies

USP dissolution-II apparatus was used to perform the powder dissolution of DS, DF, PM, and solid dispersions (DSSD and DFSD). DS and DF of 20 mg or a DS equivalent product to 20 mg of solid dispersions were utilized in the dissolution studies. The studies were performed in USP phosphate buffer (pH 6.8) of 900 mL at 37°C with a paddle speed of 75 rpm. Samples of one mL were withdrawn at time intervals of 15, 30, 60, 90, and 120 minutes and then centrifuged using a Remi C24 centrifuge for 10 minutes at 10000 rpm and 4°C. The collected supernatant was diluted and analyzed using HPLC.

#### 2.2.8. *In Vitro* Permeability Studies

Franz diffusion cell apparatus was used to carry out diffusion studies of DS, DF, and prepared solid dispersions using a semipermeable dialysis membrane of 3.5 kDa molecular weight cutoff with a 3.8 cm^2^ surface area. Franz diffusion cell apparatus assembly consists of two compartments, one donor compartment and the other receiver compartment. The donor compartment was filled with the solution of DS, DF, and their solid dispersions (DSSD and DFSD), 3 mL each, one solution at a time. The receiver compartment was loaded with 15 mL of USP phosphate buffer (50 mM) (*pH* 6.8) and agitated through a magnetic stirrer. Samples were withdrawn at regular time intervals of 0, 10, 20, 30, 40, 50, 60, 90, and 120 minutes from the receiver compartment and analyzed using HPLC.

#### 2.2.9. Pharmacokinetic Studies

Pharmacokinetic experiments were performed following GLP and CPCSEA guidelines and were approved by the Institutional Animal Ethics Committee (IAEC/KMC/22/2021), Manipal Academy of Higher Education (MAHE). *In vivo*, pre-clinical studies were conducted for DS, DF, and DSSD and DFSD. Overnight fasted male Wistar rats weighing 200 to 250 g were separated into four groups, each consisting of three rats. DTG at a dose of 10 mg/kg and solid dispersions (DSSD and DFSD) with an equivalent dose of 10 mg/kg DTG were administered in the form of suspension prepared using 0.5% sodium carboxymethyl cellulose through oral gavage. The daily maximum human dose of DTG 100 mg animal dose was calculated using the following formula along with the contemplated literature [35, 36].

(1) $$\begin{array}{c} AED\;\left(\frac{mg}{kg}\right)=human\;dose\;\left(\frac{mg}{kg}\right)\times K_{m}ratio\text{,} \end{array}$$

where *K*_*m*_ ratio for rat = 6.2, human dose of DTG = 1.6 (mg/kg), and AED = animal equivalent dose.

(2) $$\begin{array}{c} AED\;\left(\frac{mg}{kg}\right)=1.6\times 6.2=9.92\;\left(\frac{mg}{kg}\right)\text{.} \end{array}$$

Blood was collected at regular intervals from the retro-orbital vein under diethyl ether anesthesia using heparinized capillaries into Eppendorf tubes containing 20 *μ*L of 10% EDTA (IACUC 2019). The collected blood was centrifuged for 10 minutes at 10,000 rpm. The plasma obtained was stored at -80°C until analysis. Phoenix WinNonlin software of version 8.0 was employed to calculate the pharmacokinetic parameters.

## 3. Results and Discussion

### 3.1. Assay of DSSD and DFSD

**Figure 1:**
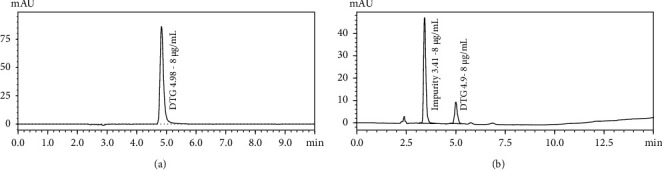
(a) Chromatogram of Dolutegravir sodium before quench cooling of 8 *μ*g/mL concentration. (b) Chromatogram of quench-cooled pure Dolutegravir sodium and its impurity of 8 *μ*g/mL concentration.

The drug content of DSSD and DFSD was determined using the developed HPLC method. In the solid dispersion samples, DSSD had a drug content of 76.71% w/w, while DFSD had a drug content of 92.21% w/w. The drug content analysis revealed that the DFSD had a higher concentration of DTG than the DSSD. This remark pertains to the temperature stability of the salt form. In addition, the thermal stability of DS was investigated by quenching pure crystalline DS and performing the assay. The drug content of quench-cooled pure DS was determined to be 41%, confirming DS degradation. To emphasise impurity, a concentration of 8 g/mL of DS before and after quench cooling was analysed using HPLC. The impurity was observed at the retention time of 3.4 min in quench-cooled DS, unlike in pure DS chromatogram (Figures 1(a) and 1(b)). Therefore, salt forms may not be a feasible choice in the preparation of ASSDs, particularly with the quench cooling method, due to their higher melting point.

### 3.2. Solid State Characterization

#### 3.2.1. DSC

**Figure 2:**
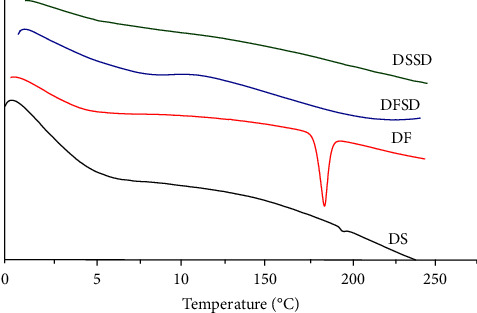
DSC thermograms of DS, DF, DFSD, and DSSD.

Pure amorphous salt DS thermogram demonstrated a glass transition temperature (Tg) at 197°C, which agrees with the literature [37, 38]. In contrast, the free acid form DF showed an endotherm at 178°C. Crystalline nature was observed for free acid from DF. DFSD and DSSD thermograms did not show any melting endotherms. As there are no sharp endotherms, we may consider it amorphous, but further characterization is required to confirm the same. Further, a material must have a characteristic Tg to be classified as DSC amorphous [39]. The Tg is not evident in both DFSD and DSSD thermograms. The thermograms of DS, DF, DFSD, and DSSD are depicted in Figure 2.

#### 3.2.2. FTIR

**Figure 3:**
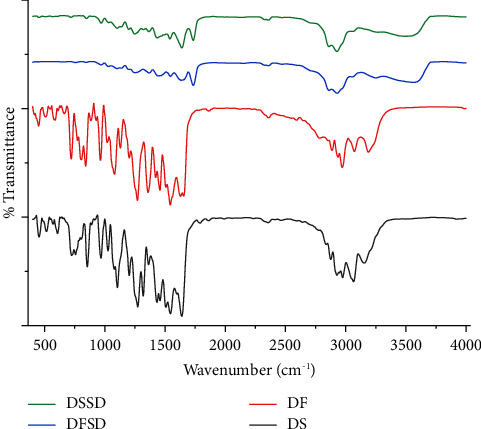
FTIR spectra of DS, DF, DFSD, and DSSD.

FTIR analysis was conducted to determine intermolecular interactions between DS, DF, and SLP. The DS and DF IR spectra were devoid of an OH stretching characteristic peak in the 3700−3584 cm^−1^ region, confirming free hydroxyl groups' absence in Figure 3. Interestingly, the broad hydroxyl band at 3550−3200 cm^−1^ in the DS and DF IR spectra corroborates the presence of intramolecular hydrogen bonding within DTG molecules [40]. Further, a peak at 1640 cm^−1^ represents the carbonyl stretching specific to secondary amide in an open chain scaffold. The spectra of free acid DF exhibited a similar pattern to salt form DS except for the shift in the carbonyl group to lower wavenumber from 1640 to 1630 cm^−1^. Except for the fact that the peaks in the dispersions are generally wider and attenuated due to the conversion of the amorphous phase, DSSD and DFSD did not demonstrate any notable shift in the peaks when compared to pure salt DS and free acid DF forms. Hence, no intermolecular interactions were observed between the DS/DF and SLP.

#### 3.2.3. XRPD

**Figure 4:**
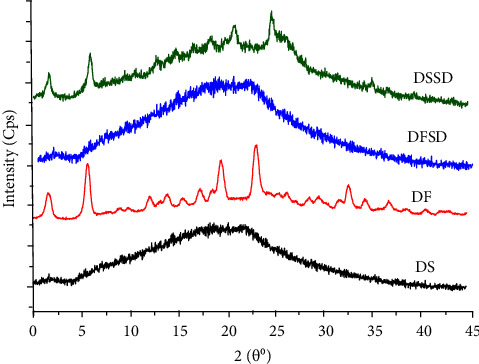
X-ray diffractograms of DS, DF, DFSD, and DSSD.

X-ray diffraction analysis confirmed the conversion of crystalline drugs into an amorphous form. Strong Bragg's diffraction was evident in the X-ray diffractogram of DF, indicating its crystalline character. DS and DFSD's diffractograms were devoid of diffraction, corroborating their amorphous nature. However, the salt solid dispersion DSSD exhibited partial crystallinity, which is apparent by diffraction peaks from its diffractogram (Figure 4). The XRPD results appear to contradict the DSC results since none of the DSC thermograms of the prepared DSSD or DFSD showed any endotherm, which is typical for the crystalline form of the drug. These conflicting results could be attributed to the DSC technique's instinctive limitation, where small traces of crystallinity were not detected within the material in many cases. Further, this result was in agreement with the observation of Jain et al. [41] who also reported the absence of the endothermic peak in DSC of Raltegravir S-SMEDDS with MCC and neusilin® US2. The crystallinity of salt solid dispersion DSSD was calculated using the following formula [36]:

(3) $$\begin{array}{c} \%\;amorphous\;content=100\times \left(1-\frac{Ap}{Ac}\right)\text{,} \end{array}$$

where Ap = cumulative Bragg's intensity of moisture samples and Ac = cumulative Bragg's intensity of PM.

The amorphicity percentage for DSSD (54%) may be ascribed to the limited miscibility of salt form DS with SLP. This indicates that the quench cooling method was ineffective in inducing disorder within the DS crystal lattice, rendering it completely amorphous.

### 3.3. Drug-Polymer Miscibility

Miscibility in ASSDs comprising a drug-polymer system is characterized as the single-phase amorphous phase formation by drug-polymer mixing. Theoretically, miscibility was calculated for DS, DF, and SLP using the Hildebrand and Hansen solubility parameters. According to the data presented in Table 1, the Hildebrand solubility parameter difference between DS or DF and SLP is more than 7 (MPa^1/2^), which implies partial miscibility. Further, the DS/DF-SLP affinity towards hydrogen bonding and dispersibility are very similar. In contrast, polar bonding affinity with SLP was higher in the case of free acid than amorphous salt. Therefore, the miscibility of DS/DF-SLP solid dispersions can be considered to some extent.

The failure of DSSD to convert into a complete amorphous form can be attributed to various factors. Primarily, the failure was due to the higher melting point of salt form DS than the free acid form DF which is evident from the respective DSC thermograms. Moreover, the excessive enthalpy of amorphous salt form DS is lesser (−0.38 J/g) compared to free acid form DF (−49.71 J/g). Therefore, the higher excessive enthalpy for free acid form DF favored the formation of a complete amorphous system, unlike salt form DS. Moreover, the drug-polymer miscibility also depends on the method employed to fabricate solid dispersions. Jog et al. prepared the solid dispersions of model compound ABT-102 using serial dilution, spray drying, and solvent evaporation. Spray-dried solid dispersions of ABT-102 demonstrated stronger miscibility between drug and polymer with higher Gibb's free energy, resulting in a more significant extent of melting point depression compared to ASDs of solvent evaporation and serial dilution [20].

### 3.4. Solubility Studies

**Figure 5:**
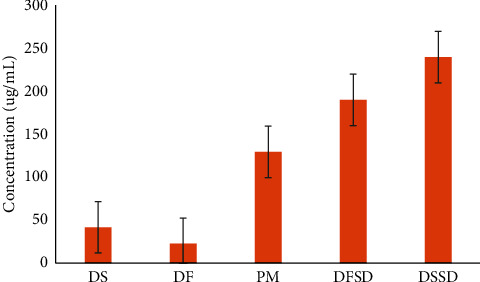
Equilibrium solubility of DS, DF, PM, DFSD, and DSSD solid dispersions in USP phosphate buffer pH 6.8.

The equilibrium solubility for salt form DS, free acid form DF, salt solid dispersion DSSD, and free acid solid dispersion DFSD was evaluated in USP phosphate buffer pH 6.8 (Figure 5). It is remarkable to note that DTG solubility is higher in the case of salt solid dispersion, which is 5.7 fold and 10.5 fold compared to pure salt as well as free acid form. Further, free acid solid dispersion DFSD also showed improvement in the DTG solubility by 4.54 and 8.3 fold, respective to their individual compounds. There are two possible rationales for the higher enhancement in solubility of DTG by salt solid dispersion DSSD than free acid solid dispersion DFSD. First, salt form improves molecule solvation by enabling dissociation into ions. Ion-dipole interactions between these ions and water molecules are more energetically favorable compared to nonionized drug and water hydrogen bonding interactions [13]. Second, converting crystalline form to an amorphous solid also results in higher solubility [42]. The solubility of DTG was improved by solid dispersions irrespective of salt and free acid forms.

### 3.5. *In Vitro* Dissolution Studies

**Figure 6:**
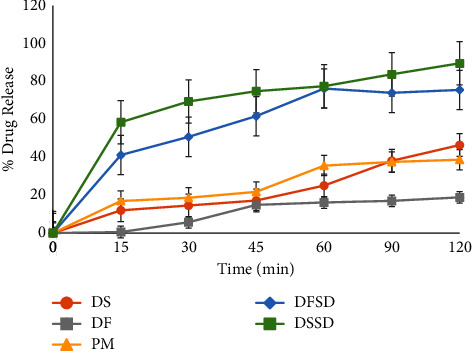
Dissolution profiles of pure DS, DF, PM, DFSD, and DSSD in USP phosphate buffer (pH 6.8).

The dissolution profiles of salt form DS, free acid form DF, and solid dispersions (DSSD and DFSD) are displayed in Figure 6. The rate and extent of dissolution were substantially greater for DSSD and DFSD compared to crystalline salt and free acid forms of DTG. The salt solid dispersion DSSD exhibited a 2-fold and 4-fold improvement in the drug release of DTG than pure salt and free acid forms. Similarly, free acid solid dispersion DFSD showed 1.5-fold and 4-fold enhancement in the DTG drug release. Owing to the rapid dissolution of the drug from DSSD and DFSD, the initial spring phase was achieved for 15 minutes in ASDs. The highest drug release obtained after 120 minutes was 89.59% for DSSD and 75.53% for DFSD.

Further, unlike pure salt and free acid forms, the generated supersaturation was maintained (parachute) in the case of both DSSD and DFSD. This phenomenon is ascribed to the inhibition of solution-mediated crystallization by the polymer in the ASDs. The dual effect of amorphization and the presence of salt form can be attributed to the higher drug release of salt dispersion DSSD than free acid DFSD in dissolution. Since the crystal lattice barrier is depleted in the amorphous form, the dissolution rate increases; additionally, the salt form impacts the self-buffering phenomenon, increasing the dissolution rate [43, 44]. The salt form modifies the pH of the proximal microenvironment in the unstirred water layer around the dissolving solid, stimulating ionization and, thus, dissolution. As a result, the self-buffering capabilities of salt drive rapid dissolution compared to free acid or base and enable improved dissolution [42]. Nevertheless, DSSD and DFSD showed enhancement in the DTG drug release irrespective of the forms than the pure crystalline phases.

### 3.6. *In Vitro* Permeability Studies

**Figure 7:**
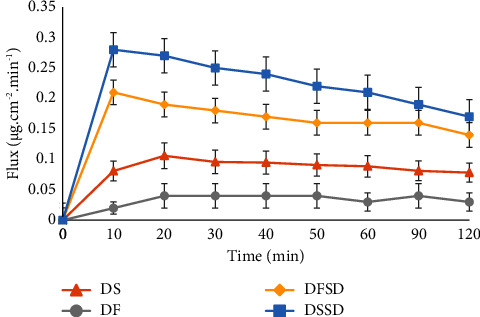
*In vitro* flux profiles of pure DS, DF, DFSD, and DSSD.

The permeability of a drug is an important factor in determining its bioavailability. Although DTG is a BCS class II candidate with high permeability, ASDs are expected to improve dissolution by generating a colloidal system [45–47]. It is imperative to study the flux of prepared DSSD and DFSD due to micelle aggregation over a period, as the colloidal system is dynamic in terms of particle size variation. Moreover, such particle size variation would considerably impact the flux over physiologically relevant time scales.  Figure 7 presents the permeability profiles of DS, DF, DFSD, and DSSD as a time function. Salt solid dispersion DSSD demonstrated the highest flux, followed by free acid dispersion DFSD. The flux of DSSD and DFSD is significantly higher than that of pure salt and free acid forms. The maximum achievable flux is determined by the amount of drug molecularly dissolved in the media [48, 49]. In this context, the flux and dissolution results agree with each other, where DSSD showed enhancement in permeability due to improved solubility than DFSD.

### 3.7. Pharmacokinetic Studies

**Figure 8:**
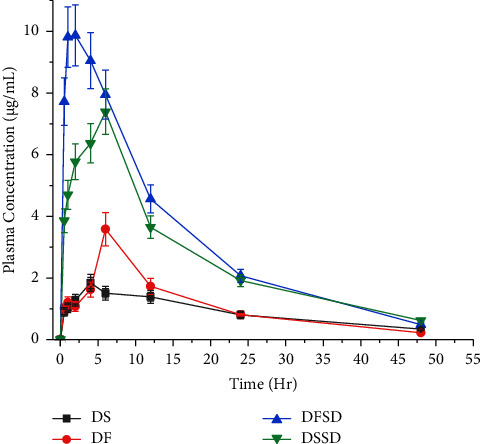
Oral bioavailability profiles of DS, DF, DFSD, and DSSD.

*In vivo* oral bioavailability study of DS, DF, DSSD, and DFSD was carried out with male Wistar rats in the fasted state. The pharmacokinetic profiles were obtained using Phoenix WinNonlin software version 8.0, employing noncompartmental analysis. The plasma drug concentration-time profiles of DS, DF, DFSD, and DSSD are depicted in Figure 8, whereas Table 2 consists of its respective pharmacokinetic parameters. At 6 hours, DTG exhibited the maximum plasma concentration (Cmax) in DS and DF of 1.84 ± 3.50 and 3.58 ± 3.94 *μ*g/mL. The prepared solid dispersions, irrespective of forms, manifested higher *C*_max_ than pure DS and DF. In particular, free acid solid dispersion DFSD improved *C*_max_ by 5.6 and 2.8 fold, followed by DSSD of 4.0 and 2.0 fold than the pure DS and DF crystalline forms. Similarly, the extent of absorption (AUC) improvement has also displayed the same pattern in ASSD. DFSD showed the highest improvement in AUC (3.9 and 3.0 fold) succeeded by DSSD (3.2 and 2.5 fold) compared to AUC of pure DS and DF.

Interestingly, *T*_max_ of free acid solid dispersion was reduced to 2 hr, unlike salt solid dispersion DSSD of 6 hr. This behavior may be attributed to the rapid absorption of the drug from amorphous solid dispersion owing to higher concentrations of molecularly dissolved API which facilitated drug-rich particle diffusion through the unstirred layer. In the case of DSSD, the lack of a complete amorphous form limited the generation of drug-rich phase particles; thereby, *T*_max_ of DSSD is analogous to the pure DLT. The shorter *T*_max_ of DFSD may be advantageous regarding the rapid onset of action after having the formulation and also the reduction of dose required to show therapeutic action. Statistical analysis was carried out across the groups using one-way ANOVA (Dunnett's test) with GraphPad Prism version 8.0. DSSD and DFSD showed a statistically significant difference (*p* value <0.05) from pure DS and DF forms. The pharmacokinetic results are not incongruent with the *in vitro* dissolution profiles of DSSD and DSFD in USP phosphate buffer (pH 6.8).

There are two possible rationales for the marginal dissimilarity of the DSSD *in vivo* profile compared to the *in vitro* dissolution profile. First, the dissolution media composition in this study is different from the intestinal fluid composition. Specifically, USP phosphate buffer (pH 6.8) was devoid of bile salts and thus did not precisely correlate with the *in vivo* profile. Bile salts and the pH of the media are key factors that govern the dissolution rate of compounds in the GIT. A case study on phenytoin was performed to observe the effect of dissolution media on drug release. Compendial and bio-relevant media (SGF, FaSSIF, FeSSIF, Blank FaSSIF, and Blank FeSSIF) were used as dissolution media. The *in vitro* dissolution profiles from bio-relevant media correspond to the *in vivo* profiles of Phenytoin, unlike the profiles of compendial media [50]. Hence, to specifically mimic the *in vivo* performance of a drug using a dissolution method, the dissolution medium must be able to simulate the GIT environment. The human GIT environment is complex, with pH variations as well as the presence of solubilizing bile salts and phospholipids along the GIT [51, 52]. Second, DSSD was not completely converted to an amorphous form, as partial crystallinity was observed from XRPD. He et al. prepared solid dispersions of Curcumin using PVP, HP-*β*-CD, and poloxamers by the solvent evaporation method. Further, HP-*β*-CD-based solid dispersions showed the highest improvement in drug release of Curcumin from dissolution profiles which did not translate to the *in vivo* bioavailability. This is due to the lack of conversion of HP-*β*-CD solid dispersion to a completely amorphous form [53]. Nevertheless, DSSD and DFSD improved the bioavailability of DTG when compared to the pure salt and free acid forms.

## 4. Conclusion

In this study, ASSDs of DTG salt (DSSD) and free acid form (DFSD) were formulated with SLP using the quench cooling method. DSC, XRPD, and FTIR characterized the prepared ASDs. XRPD revealed the failure of the quench cooling method to induce a completely amorphous form for DSSD salt solid dispersion, unlike free acid dispersion. Further, the DSSD and DFSD exhibited improved solubility and dissolution irrespective of salt and free acid dispersions compared to their crystalline salt and free acid counterparts. The enhanced dissolution of DSSD and DFSD was also manifested in the improved flux. Nevertheless, due to partial crystallinity, the dissolution advantage of salt solid dispersion DSSD was insignificant in the oral bioavailability study in male Wistar rats. The free acid solid dispersion DFSD demonstrated a significant improvement in the plasma concentration of DTG compared to DSSD.

Further future experiments will be attempted including solvent evaporation, hot-melt extrusion, ball milling, and spray drying for complete amorphous salt solid dispersion. *In situ* amorphization is another technique for preparing amorphous salts using spray drying. In this experiment, the drug, counterions, and polymer are dissolved in solvents like methanol and subjected to mixing for a homogenous solution. The final solution was spray-dried to attain amorphous salts. In the case of ball milling, drugs and salts like succinic acid and citric acid are mixed in various molar ratios and ground for a few hours. Later the polymer in a selected or optimized ratio of w/w is added to the obtained drug organic acid mixture and ground for a few minutes to get the amorphous salt solid dispersion.

## Figures and Tables

**Table 1:** Drug coformer miscibility calculation using Hildebrand and Hansen solubility parameter method.

S.no	Substance	Molecular weight (g/mol)	Density (g/cm^3^)	Molecular volume (cm^3^/mol)	Melting point (K)	Solubility parameter (MPa^1/2^)			
						Hildebrand	Hansen		
							*δd*	*δp*	*δh*
1	Dolutegravir sodium	441.36	0.9	490.4	—	33.18	13.5	8.2	10.8
2	Dolutegravir	419.38	0.9	465.98	454.15	32.17	12.5	7.2	10.3
3	Soluplus	118000	1.20	98333	—	24.87	17.4	0.3	8.6

**Table 2:** Pharmacokinetic parameters after a single oral dose equivalent to 10 mg/kg of DS, DF, DSSD, and DFSD (*n* = 3).

S.no	Parameter	DS	DF	DFSD	DSSD
1	AUC_0−*t*_ (*μ*g·h/mL)	39.17 ± 5.16	51.17 ± 8.23	156.05 ± 10.15^*∗*^	128.29 ± 13.45^*∗*^
2	*C*_max_ (*μ*g/mL)	1.84 ± 3.50	3.58 ± 3.94	10.38 ± 20.77^*∗*^	7.42 ± 15.25^*∗*^
3	*T*_max_ (hr)	6	6	2	6
4	*t*½ (hr)	13.91	12.69	11.43	14.33

^∗^ indicates a significant difference when compared to DS and DF. ^∗^*p* < 0.05 significantly different.

## Data Availability

The data used to support the findings of this study are included within the article.

## Supplementary Materials

Supplementary Materials This section consists of Dolutegravir sodium amorphous salt, Dolutegravir free acid, and Raloxifene structures. Further, bioanalytical method validation parameters include system suitability test, specificity, linearity, accuracy and precision, carry over check, recovery, stock solution stability, and long-term stability data. It also consists of Blank and LLOQ representative chromatograms for the DTG and RLX extracted from the plasma samples.
