# Supplementary material for: Exploring the Solubility and Bioavailability of Sodium Salt and Its Free Acid Solid Dispersions of Dolutegravir
Source: Adv Pharmacol Pharm Sci. 2023 Jun 20;2023:7198674. doi: 10.1155/2023/7198674 (PMC10299877; doi:10.1155/2023/7198674)
Supplement: Supplementary Materials — This section consists of Dolutegravir sodium amorphous salt, Dolutegravir free acid, and Raloxifene structures. Further, bioanalytical method validation parameters include system suitability test, specificity, linearity, accuracy and precision, carry over check, recovery, stock solution stability, and long-term stability data. It also consists of Blank and LLOQ representative chromatograms for the DTG and RLX extracted from the plasma samples. [file 7198674.f1.docx]

Figure. 1a Chemical Structure of Dolutegravir Sodium

Figure. 1b Chemical Structure of Dolutegravir free acid

Figure. 1c Chemical Structure of Raloxifene

**Bioanalytical method validation parameters:**

DTG : Dolutegravir; RLX: Raloxifene HCl; CV : Coefficient of variation; SD : Standard deviation; RT : Retention time; IS : Internal Standard

**System suitability test:**

|  | Injection | | | | | Mean | SD | %CV | Inference |
| --- | --- | --- | --- | --- | --- | --- | --- | --- | --- |
|  | 1 | 2 | 3 | 4 | 5 |  |  |  |  |
| Peak area ratio | 6.54 | 6.52 | 6.55 | 6.50 | 6.44 | 6.51 | 0.04 | 0.64 | pass |
|  |  |  |  |  |  |  |  |  |  |
| RT of DTG | 11.11 | 11.11 | 11.11 | 11.11 | 11.11 | 11.11 | 0.00 | 0.03 | pass |
|  |  |  |  |  |  |  |  |  |  |
| RT of RLX | 6.19 | 6.20 | 6.20 | 6.19 | 6.20 | 6.19 | 0.00 | 0.04 | pass |
|  |  |  |  |  |  |  |  |  |  |

**Acceptance criteria:** % Coefficient of variation for peak area ratio must be ≤ 2.0%.

**Specificity:**

| Mean extracted LLOQ area=24574  20% area of LLOQ=4914.8 | | | | |
| --- | --- | --- | --- | --- |
| Mean extracted IS area=45769.5  5% area of Internal Standard=2288.5 | | | | |
| Sample | Interference at RT of DTG | Inference | Interference at RT of RLX (IS | Inference |
|  |  |  |  |  |
| Blank plasma | 1838 | pass | - | pass |
|  | 2000 | pass | - | pass |
|  | 1366 | pass | - | pass |
|  | 2268 | pass | - | pass |
| Zero sample | - | pass | 47121 | - |
|  | - | pass | 45308 |  |
|  | - | pass | 45286 |  |
|  | - | pass | 45363 |  |
| LLOQ | 24443 | - | 44377 | - |
|  | 24297 |  | 44949 |  |
|  | 24545 |  | 45005 |  |
|  | 25011 |  | 45003 |  |

**Acceptance criteria:**

- There should be no interference within ± 10% window of analyte and internal standard.
- If there is any interference it must be less than 20% of LLOQ area of analyte and ≤5% of internal standard

**Linearity:**

|  | Back calculated Conc. (µg/mL) | | | Mean | % Nominal Conc. | SD | % CV | Inference |
| --- | --- | --- | --- | --- | --- | --- | --- | --- |
| Conc. (µg/mL) | Batch 1 | Batch 2 | Batch 3 |  | | | |  |
| 0.35 | 0.3 | 0.36 | 0.33 | 0.33 | 94.29 | 0.03 | 9.09 | Pass |
| 0.7 | 0.7 | 0.71 | 0.73 | 0.71 | 101.9 | 0.02 | 2.14 | Pass |
| 1.4 | 1.5 | 1.33 | 1.42 | 1.42 | 101.19 | 0.09 | 6.00 | Pass |
| 2.8 | 2.71 | 2.87 | 2.72 | 2.77 | 98.81 | 0.09 | 3.24 | Pass |
| 5.6 | 5.71 | 5.61 | 5.67 | 5.66 | 101.13 | 0.05 | 0.89 | Pass |
| 7.0 | 6.94 | 6.98 | 6.99 | 6.97 | 99.57 | 0.03 | 0.38 | Pass |

**Acceptance criteria**:

- Nominal concentration of back calculated concentration should be between 80-120% for STD-1 and 85-115% for other standards (STD 2-5)

**Accuracy and Precision**

1. **Intra-batch or within-batch Precision & Accuracy**

|  | LLOQ (0.35 µg/mL) | | LQC (1.05 µg/mL) | | MQC (3.5 µg/mL) | | HQC (6.3 µg/mL) | |
| --- | --- | --- | --- | --- | --- | --- | --- | --- |
| S.No. | Calculated Conc. | % Nominal | Calculated Conc. | %  Nominal | Calculated Conc. | % Nominal | Calculated Conc. | %  Nominal |
| 1 | 0.3 | 85.71 | 1.06 | 100.95 | 3.46 | 98.86 | 6.17 | 97.94 |
| 2 | 0.31 | 88.57 | 1.12 | 106.67 | 3.64 | 104.00 | 6.38 | 101.27 |
| 3 | 0.28 | 80.00 | 1.01 | 96.19 | 3.35 | 95.71 | 5.88 | 93.33 |
| 4 | 0.3 | 85.71 | 1.02 | 97.14 | 3.22 | 92.00 | 6.08 | 96.51 |
| 5 | 0.36 | 102.86 | 1.05 | 100.00 | 3.2 | 91.43 | 6.02 | 95.56 |
| 6 | 0.35 | 100.00 | 0.97 | 92.38 | 3.36 | 96.00 | 6.06 | 96.19 |
| Mean | 0.32 | 90.48 | 1.04 | 98.89 | 3.37 | 96.33 | 6.10 | 96.80 |
| %CV | 9.92 |  | 4.93 |  | 4.84 |  | 2.74 |  |

1. **Inter batch or between batch precision and accuracy.**

| Conc. (µg/mL) | LLOQ(0.35 µg/mL) | LQC | MQC | HQC |
| --- | --- | --- | --- | --- |
|  |  | (1.05 µg/mL) | (3.5 µg/mL) | (6.3 µg/mL) |
| Number | 18 | 18 | 18 | 18 |
| Mean | 0.32 | 1 | 3.25 | 5.98 |
| SD | 0.02 | 0.03 | 0.12 | 0.1 |
| %CV | 7 | 4.35 | 3.52 | 1.63 |
| % Nominal Conc. | 90 | 95.66 | 92.73 | 94.92 |

**Acceptance criteria:**

- % Nominal concentration of back calculated concentration at QC level should be between 85-115%, except for the LLOQ 80-120%

**Carry over check:**

| 20% area of LLOQ=3911 | | | | |
| --- | --- | --- | --- | --- |
| 5% area of IS =2239 | | | | |
| S.No | Sample | Area | | Inference |
|  |  | DTG | RLX |  |
| 1 | Blank solution | 613 | 0 | pass |
| 2 | Un-extracted ULOQ | 630752 | 43894 | pass |
|  |  |  |  |  |
| 3 | Blank solution | 590 | 0 | pass |
| 4 | Un-extracted ULOQ | 628029 | 43718 | pass |
| 5 | Blank solution | 513 | 0 | pass |
| 6 | Blank plasma | 1805 | 0 | pass |
| 7 | EXT-ULOQ | 590914 | 40710 | pass |
| 8 | Blank plasma | 1083 | 0 | pass |
| 9 | EXT-ULOQ | 589538 | 40531 | pass |
| 10 | Blank plasma | 1061 | 0 | pass |

**Acceptance criteria**:

- Carry over should not exceed 20% of area of the analyte LOQ QC and should not exceed 5% of the area of the ISTD.

**Recovery:**

| S.No | LQC (1.05 µg/mL) | | | MQC (3.5 µg/mL) | | | HQC (6.3 µg/mL) | | |
| --- | --- | --- | --- | --- | --- | --- | --- | --- | --- |
|  | Unextracted Area | Extracted area | % recovery | Unextracted Area | Extracted area | % recovery | Unextracted Area | Extracted area | % Recovery |
| 1 | 76595 | 66822 | 87.24 | 292567 | 255944 | 87.48 | 564363 | 542444 | 96.12 |
| 2 | 79584 | 69273 | 87.04 | 306723 | 282949 | 92.25 | 585322 | 522556 | 89.28 |
| 3 | 80863 | 71832 | 88.83 | 316882 | 272093 | 85.87 | 594845 | 559342 | 94.03 |
| 4 | 79593 | 66829 | 83.96 | 318824 | 284738 | 89.31 | 587743 | 546968 | 93.06 |
| 5 | 79388 | 71035 | 89.48 | 329588 | 269549 | 81.78 | 584443 | 554374 | 94.86 |
| 6 | 78567 | 69679 | 88.69 | 318727 | 262259 | 82.28 | 593244 | 537525 | 90.61 |
| Mean | 79098.3 | 69245 | 87.54 | 313885 | 271255 | 86.5 | 584993 | 543868 | 92.99 |
| SD | 1430.21 | 2087.76 | 1.99 | 12721.3 | 11291.7 | 4.06 | 10942.7 | 13083.5 | 2.6 |
| %CV | 1.81 | 3.02 | 2.28 | 4.05 | 4.16 | 4.69 | 1.87 | 2.41 | 2.8 |
| Mean  % Recovery | 87.54 | | | 86.5 | | | 92.99 | | |

• Acceptance criteria: % Coefficient of Variation across QC levels must be ≤ 20

**Stability:**

**Stock solution Stability:**

| S.No | MQC (3.5 µg/mL) | | | |
| --- | --- | --- | --- | --- |
|  | DTG | | RLX | |
|  | Fresh comparison sample area | Stability  sample area | Fresh comparison  Sample  area | Stability  sample area |
| 1 | 244289 | 222289 | 45831 | 42342 |
| 2 | 249244 | 229244 | 44986 | 41346 |
| 3 | 248715 | 228715 | 44196 | 41532 |
| 4 | 251333 | 231423 | 45322 | 40642 |
| 5 | 248436 | 229543 | 44532 | 41553 |
| Mean | 248403.40 | 228242.80 | 44973.40 | 41483 |
| %CV | 1.03 | 1.53 | 1.43 | 1.46 |

Acceptance criteria: % change of DTG and RLX should be less than ± 10%

**Long term stability:**

| S.No | LQC (1.05 µg/mL) | | | |
| --- | --- | --- | --- | --- |
|  | Freshly prepared samples | | Stability samples | |
|  | Calculated. Conc. | %Nominal conc. | Calculated. Conc. | %Nominal conc. |
| 1 | 1.00 | 95.24 | 0.88 | 83.81 |
| 2 | 0.98 | 93.33 | 0.88 | 83.81 |
| 3 | 0.95 | 90.48 | 0.89 | 84.76 |
| 4 | 0.99 | 94.29 | 0.9 | 85.71 |
| 5 | 1.02 | 97.14 | 0.89 | 84.76 |
| 6 | 0.96 | 91.43 | 0.86 | 81.90 |
| Mean | 0.98 | 93.65 | 0.88 | 84.13 |
| %CV | 2.63 | | 1.55 | |
| Mean  % change | -10.17 | | | |

Acceptance criteria: % change of DTG and RLX should be less than ± 10%

All the validation parameters mentioned above are passed and within limits.

RLX / 6.223/44546

DTG / 11.196/20935

Datafile Name:LLOQ(1).lcd

Sample Name:LLOQ(1)

Sample ID:LLOQ(1)

0.0

1.0

2.0

3.0

4.0

5.0

6.0

7.0

8.0

9.0

10.0

11.0

12.0

13.0

min

0.0

0.5

1.0

1.5

2.0

2.5

3.0

mV

| Peak | Name | Ret. Time | Area | Height | Area% | Tailing F | NTP(USP) | S/N | HETP(USP) | Width(50%) | Noise |
| --- | --- | --- | --- | --- | --- | --- | --- | --- | --- | --- | --- |
| 1 | RLX | 6.223 | 44546 | 3091 | 68.029 | 1.497 | 4522 | 32.76 | 55.282 | 0.217 | 94.36 |
| 2 | DTG | 11.196 | 20935 | 968 | 31.971 | 1.575 | 7089 | 10.25 | 35.266 | 0.319 | 94.36 |

Figure 2: LLOQ representative chromatogram for the DTG and RLX extracted from the plasma samples

2.0

3.0

4.0

5.0

6.0

7.0

8.0

9.0

10.0

11.0

12.0

13.0

min

0.0

2.5

5.0

7.5

10.0

12.5

mV

Figure 3: Blank representative chromatogram extracted from the plasma samples.
